# Supplementary material for: The Effect of Glucose on the Interaction of Bisphenol A and Bovine Hemoglobin Characterized by Spectroscopic and Molecular Docking Techniques
Source: Int J Mol Sci. 2023 Sep 28;24(19):14708. doi: 10.3390/ijms241914708 (PMC10572490; doi:10.3390/ijms241914708)
Supplement: Supplementary file 1 [file ijms-24-14708-s001.zip › ijms-2589317-supplementary.pdf]

Supplementary Material

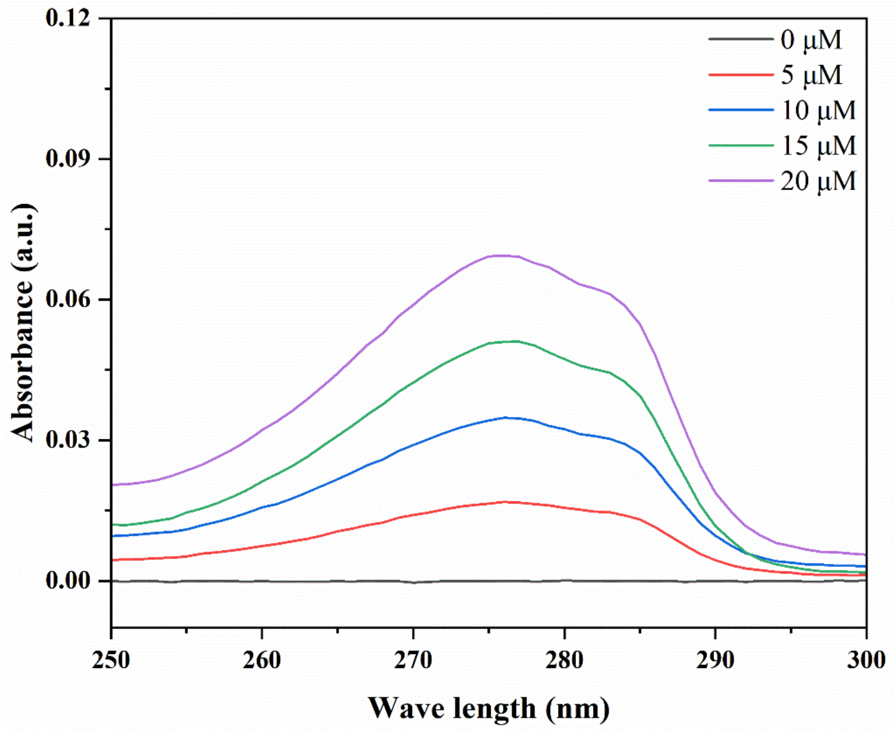

Figure S1. Absorbance of different concentrations of BPA in 0.1 M phosphate buffer.

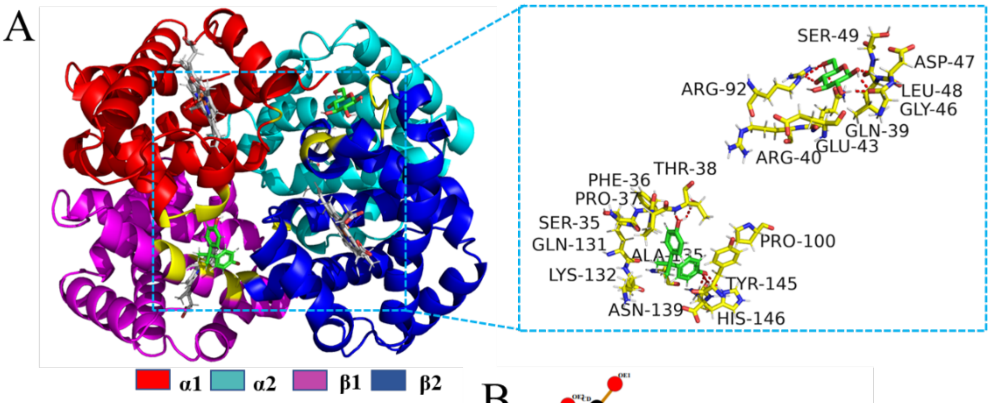
A
SER-49
ASP-47
ARG-92
LEU-48
GLY-46
GLN-39
GLU-43
ARG-40
THR-38
PHE-36
PRO-37
SER-35
GLN-131
PRO-100
LYS-132
TYR-145
ASN-139
HIS-146
α1
α2
β1
β2

1

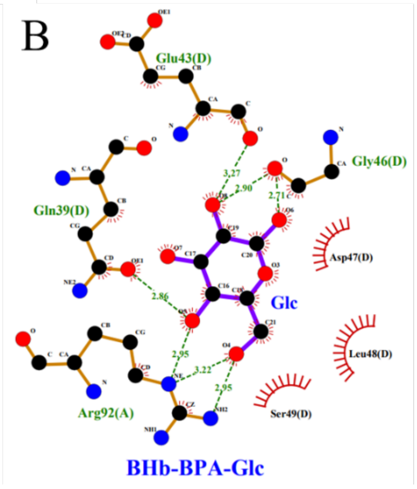
B
Glu43(D)
Gly46(D)
Gln39(D)
Asp47(D)
Glc
Leu48(D)
Arg92(A)
Ser49(D)
BHb-BPA-Glc

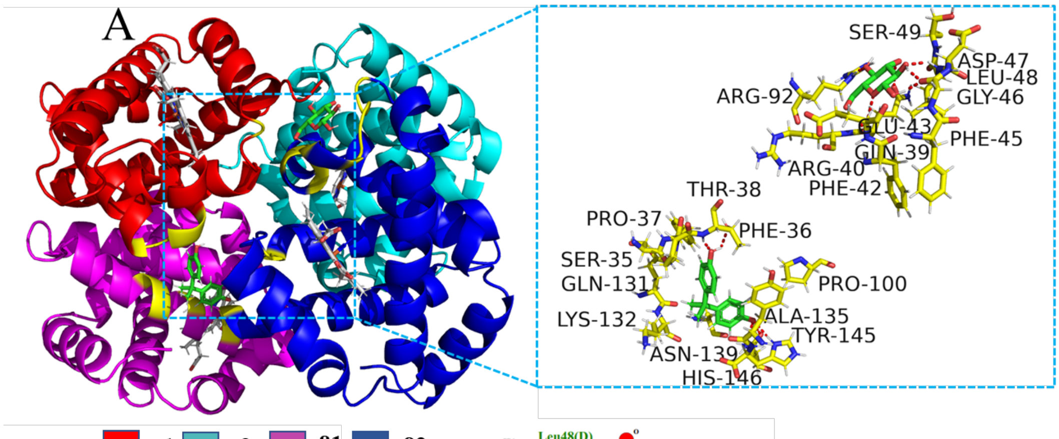
A
SER-49
ASP-47
LEU-48
ARG-92
GLY-46
GLU-43
PHE-45
GLN-39
ARG-40
PHE-42
THR-38
PRO-37
PHE-36
SER-35
PRO-100
GLN-131
ALA-135
LYS-132
TYR-145
ASN-139
HIS-146
α1
α2
β1
β2

2

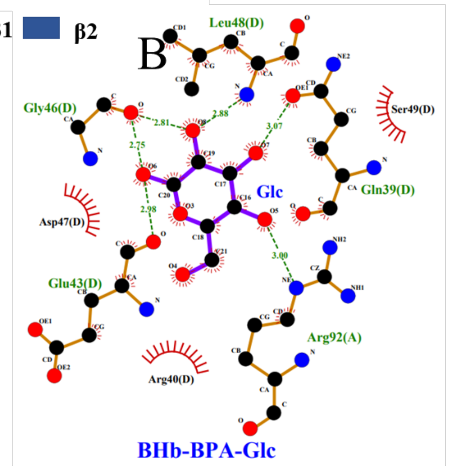
B
Leu48(D)
Gly46(D)
Ser49(D)
Glc
Gln39(D)
Asp47(D)
Glu43(D)
Arg92(A)
Arg40(D)
BHb-BPA-Glc

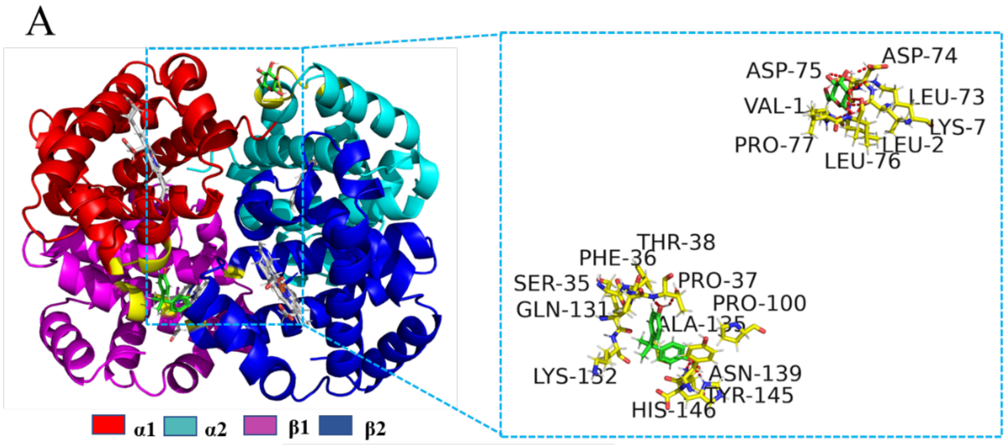
A
ASP-75
ASP-74
VAL-1
LEU-73
LYS-7
PRO-77
LEU-2
LEU-76
THR-38
PHE-36
SER-35
PRO-37
PRO-100
GLN-131
ALA-135
LYS-132
ASN-139
TYR-145
HIS-146
α1
α2
β1
β2

3

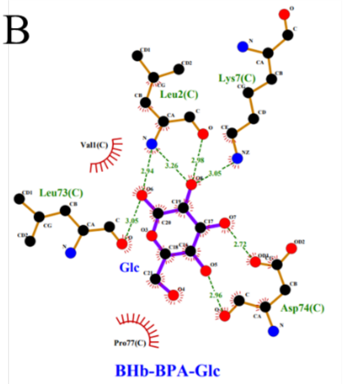
B
Leu2(C)
Lys7(C)
Val1(C)
Leu73(C)
Glc
Asp74(C)
Pro77(C)
BHb-BPA-Glc

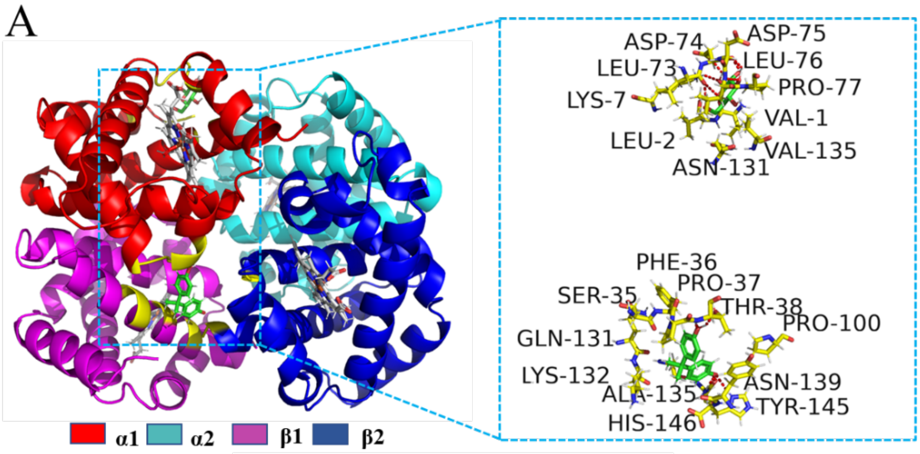
A
ASP-74
ASP-75
LEU-73
LEU-76
LYS-7
PRO-77
VAL-1
LEU-2
VAL-135
ASN-131
PHE-36
PRO-37
SER-35
THR-38
PRO-100
GLN-131
LYS-132
ALA-135
ASN-139
HIS-146
TYR-145
α1
α2
β1
β2

4

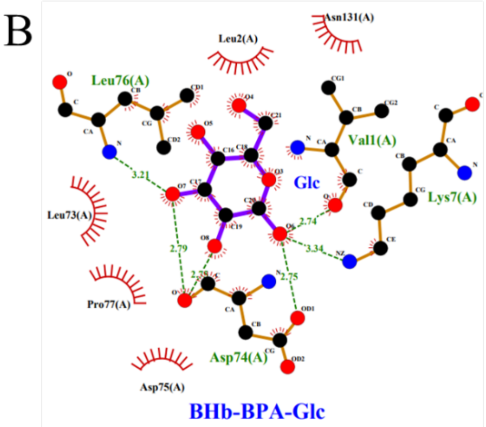
B
Asn131(A)
Leu2(A)
Leu76(A)
Val1(A)
Glc
Lys7(A)
Leu73(A)
Pro77(A)
Asp74(A)
Asp75(A)
BHb-BPA-Glc

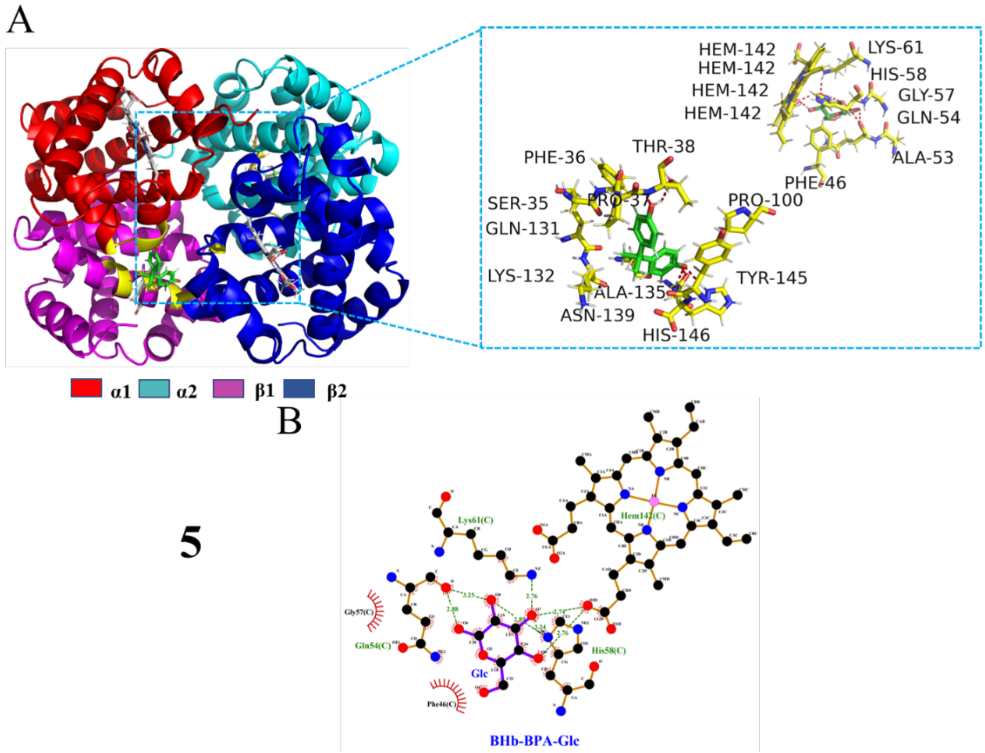
A
HEM-142
HEM-142
HEM-142
HEM-142
LYS-61
HIS-58
GLY-57
GLN-54
ALA-53
PHE-46
PHE-36
THR-38
SER-35
PRO-37
PRO-100
GLN-131
LYS-132
ALA-135
TYR-145
ASN-139
HIS-146
α1
α2
β1
β2
B
Lys61(C)
Hem142(C)
Gly57(C)
Gln54(C)
His58(C)
Glc
Phe46(C)
BHb-BPA-Glc

5

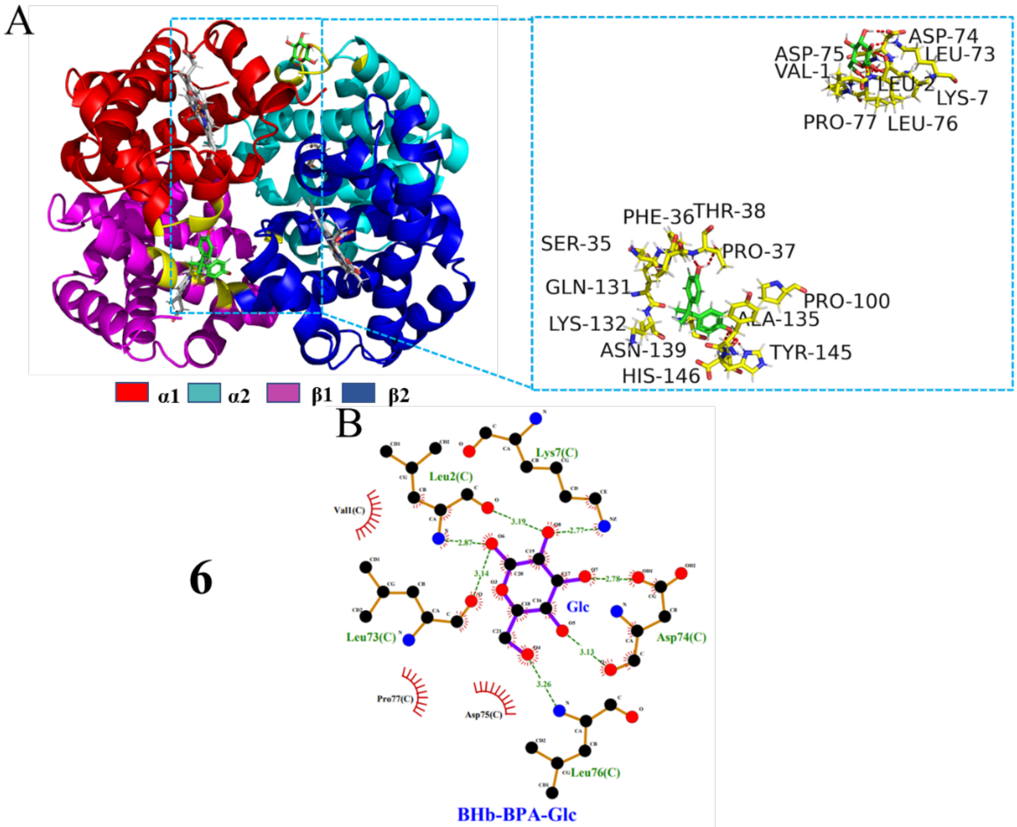
A
ASP-74
ASP-75
LEU-73
VAL-1
LEU-2
LYS-7
PRO-77
LEU-76
PHE-36
THR-38
SER-35
PRO-37
GLN-131
PRO-100
LYS-132
ALA-135
ASN-139
TYR-145
HIS-146
α1
α2
β1
β2
B
Lys7(C)
Leu2(C)
Val1(C)
Glc
Leu73(C)
Asp74(C)
Pro77(C)
Asp75(C)
Leu76(C)
BHb-BPA-Glc

6

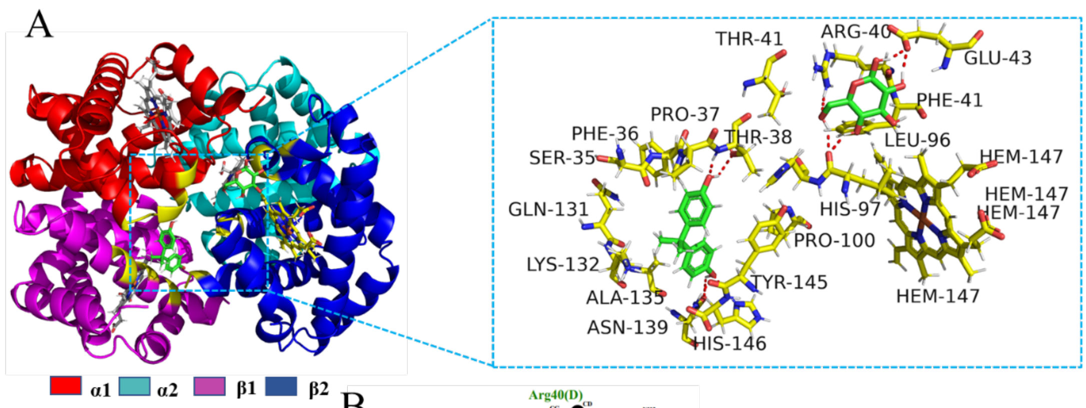
A
THR-41
ARG-40
GLU-43
PHE-41
PRO-37
PHE-36
THR-38
LEU-96
SER-35
HEM-147
HEM-147
HEM-147
GLN-131
HIS-97
PRO-100
LYS-132
TYR-145
ALA-135
HEM-147
ASN-139
HIS-146
α1
α2
β1
β2

7

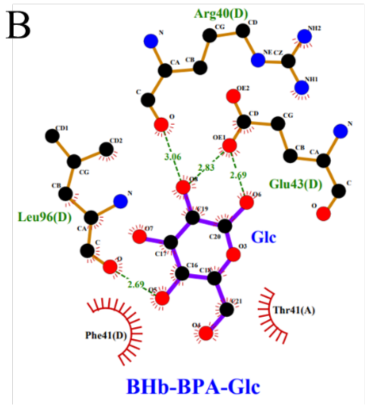
B
Arg40(D)
Glu43(D)
Leu96(D)
Glc
Thr41(A)
Phe41(D)
BHb-BPA-Glc

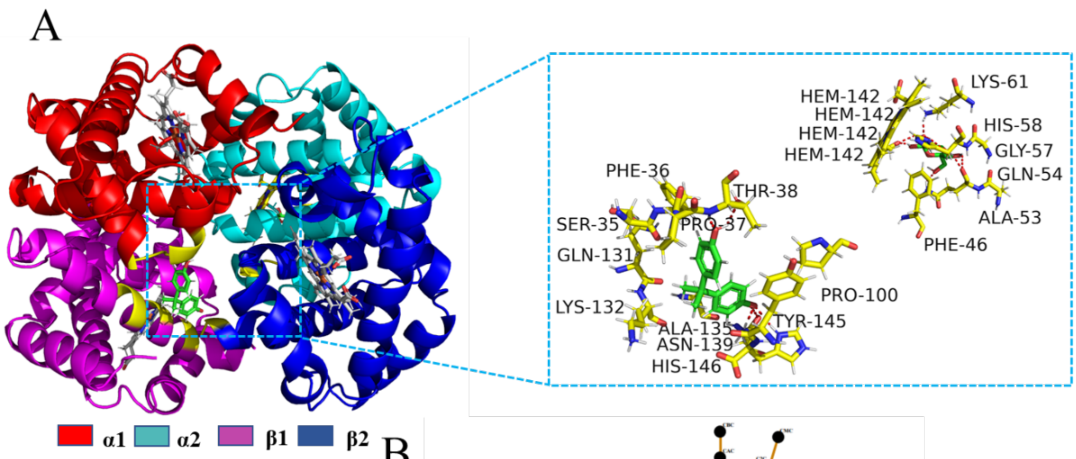
A
HEM-142
HEM-142
HEM-142
HEM-142
LYS-61
HIS-58
GLY-57
GLN-54
PHE-36
THR-38
SER-35
PRO-37
ALA-53
GLN-131
PHE-46
PRO-100
LYS-132
ALA-135
TYR-145
ASN-139
HIS-146
α1
α2
β1
β2

8

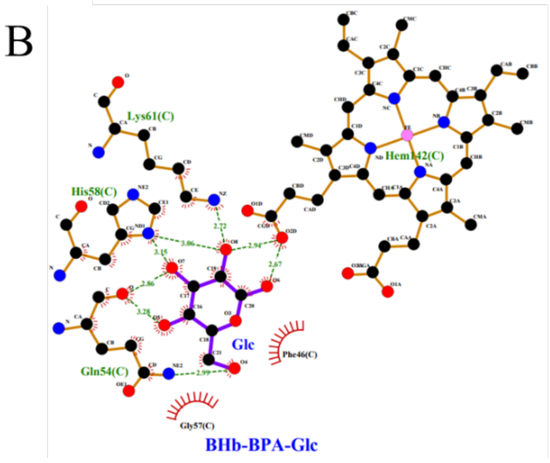
B
Lys61(C)
His58(C)
Hem142(C)
Glc
Phe46(C)
Gln54(C)
Gly57(C)
BHb-BPA-Glc

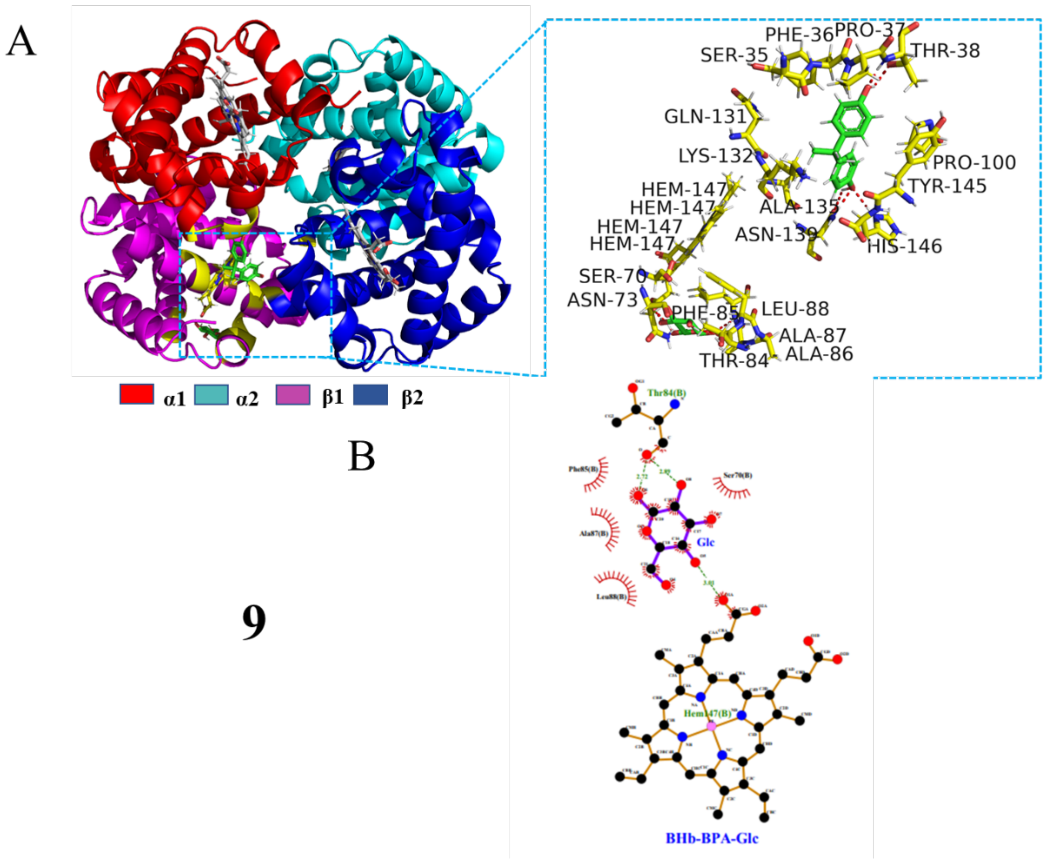
A
SER-35
PHE-36
PRO-37
THR-38
GLN-131
LYS-132
PRO-100
TYR-145
HEM-147
HEM-147
HEM-147
HEM-147
ALA-135
ASN-139
HIS-146
SER-70
ASN-73
PHE-85
LEU-88
ALA-87
ALA-86
THR-84
α1
α2
β1
β2
B
Thr84(B)
Phe85(B)
Ser70(B)
Ala87(B)
Leu88(B)
Glc
Hem147(B)
BHb-BPA-Glc

9

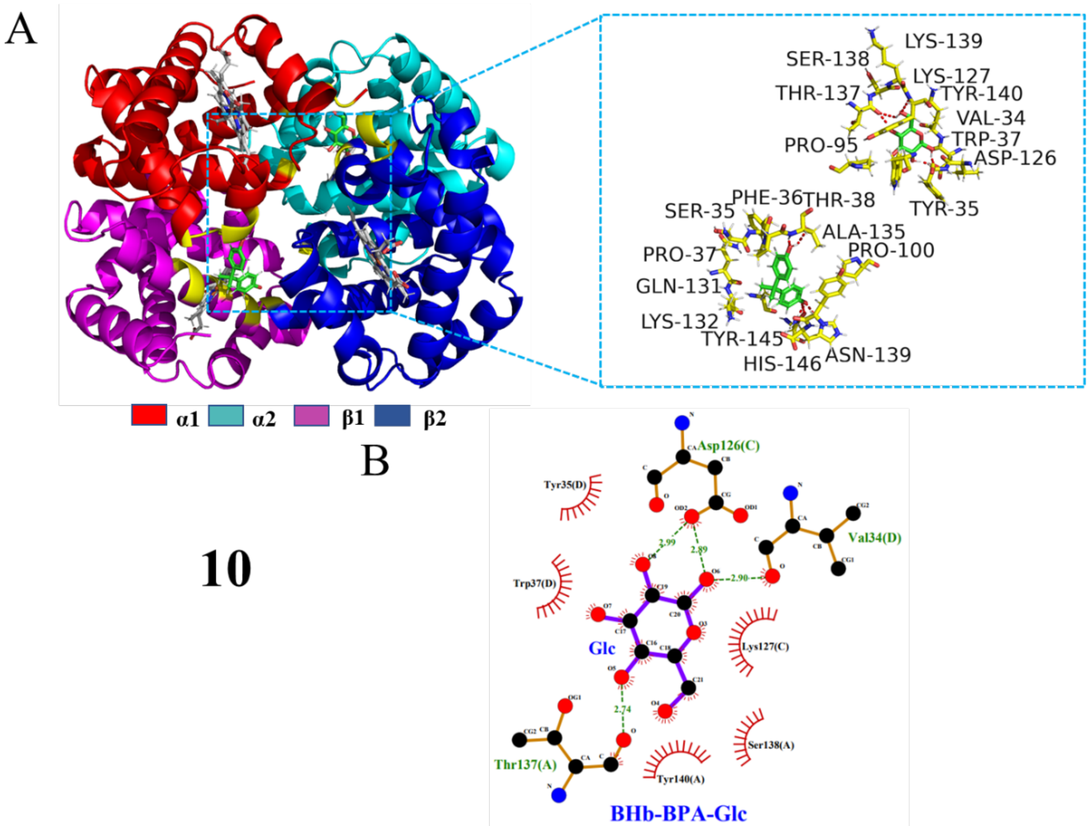
A
LYS-139
SER-138
LYS-127
THR-137
TYR-140
VAL-34
PRO-95
TRP-37
ASP-126
SER-35
PHE-36
THR-38
TYR-35
ALA-135
PRO-37
PRO-100
GLN-131
LYS-132
TYR-145
HIS-146
ASN-139
α1
α2
β1
β2
B
Asp126(C)
Tyr35(D)
Val34(D)
Trp37(D)
2.99
2.89
2.90
Glc
Lys127(C)
2.74
Thr137(A)
Ser138(A)
Tyr140(A)
BHb-BPA-Glc

10

Figure.S2. The molecular docking of Glc with BHb-BPA was ranked according to the binding energy and the top 10 docking results with the lowest binding energy were obtained.

Table S1. The top 10 lowest binding energies obtained by molecular docking of Glc with BHb-BPA.

Binding site	1	2	3	4	5	6	7	8	9	10
Binding energy (kcal)	-4.1	-3.83	-3.74	-3.44	-3.39	-3.3	-3.29	-3.24	-3.23	-3.19
